# Supplementary material for: Direct Inhibition of the Allergic Effector Response by Raw Cow’s Milk—An Extensive In Vitro Assessment
Source: Cells. 2020 May 19;9(5):1258. doi: 10.3390/cells9051258 (PMC7290799; doi:10.3390/cells9051258)
Supplement: Supplementary file 1 [file cells-09-01258-s001.pdf]

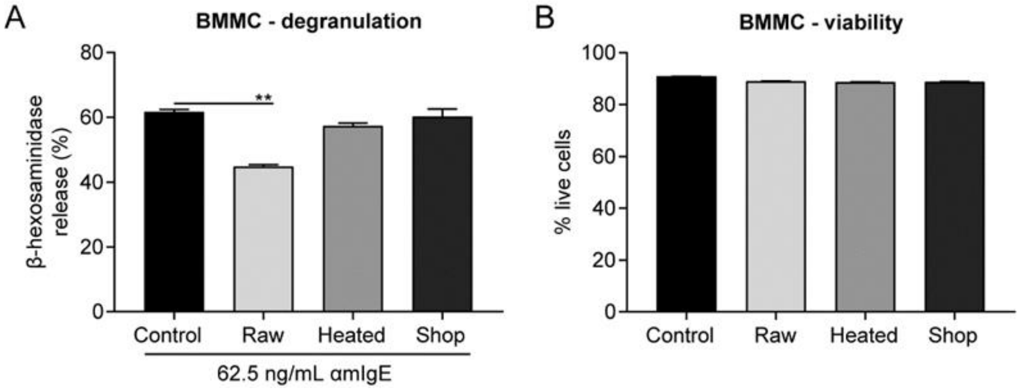

Figure S1. Mast cell activation in response to IgE cross-linking is reduced by raw milk without affecting cellular viability. Primary mouse BMMC were incubated overnight with raw milk, heated raw milk, or shop milk before they were primed with IgE and stimulated by 62.5 ng/mL rat anti-mouse IgE mAb. (**A**) β-hexosaminidase release measured in supernatant collected 1 h after αmIgE stimulation. (**B**) Viability of cultured BMMC after overnight milk exposure. Data are presented as mean ± SEM and are representative of three independent experiments. $^{**}P < 0.01$, compared to the control group as analyzed with one-way ANOVA followed by Dunnett's multiple comparisons test. BMMC, bone marrow-derived mast cells; raw, raw cow's milk; heated, heated raw cow's milk; shop, shop milk; αmIgE, rat anti-mouse IgE.
